# Piezoelectric ceramic sensor-based structural health monitoring of diagonally braced H-shaped steel structures

**DOI:** 10.1038/s41598-023-40758-6

**Published:** 2023-09-11

**Authors:** Cai Wu, Quanmao Xiao, Daopei Zhu

**Affiliations:** 1 Changjiang River Scientific Research Institute, Changjiang Water Resources Commission, Wuhan, 430010 China; 2 School of Civil Engineering, Hubei Engineering University, Xiaogan, 432000 Hubei China; 3 School of Civil and Surveying & Mapping Engineering, Jiangxi University of Science and Technology, Ganzhou, 341000 China

**Keywords:** Civil engineering, Ceramics

## Abstract

In this study, we propose a piezoelectric ceramic sensor-based health monitoring method to monitor the stress state of diagonally braced H-shaped steel structures. Loading experiments were carried out on diagonally braced H-shaped steel under different working conditions. Subsequently, the amplitude and energy of piezoelectric signals under these two working conditions were compared and analyzed, and finite element analysis was performed using ABAQUS software to verify the results. The experimental results showed that with an increase in the web height or load, the time-domain waveform energy index of the H-shaped steel increased. Under different working conditions, such as a diagonally braced H-shaped steel member with a web height of 10 cm, when the pressure value was less than 10 N/mm^2^, the energy index increased by approximately 15.98% for every 1 N/mm^2^ increase in the pressure. When the pressure value was greater than 10 N/mm^2^ and less than 15 N/mm^2^, the energy index increased by approximately 1% for every 1 N/mm^2^ increase in pressure. Further, the energy index increased by approximately 8.4% for every 1 cm increase in the height of the web. Simultaneously, it can be seen from the results of the finite element analysis that the stress and strain at the induction position of the piezoelectric ceramic sensor increased with an increase in the external pressure. The study of structural health monitoring for diagonally braced H-shaped steel structures holds significant importance in ensuring the safety and reliability of these structures, achieving predictive maintenance, evaluating structural performance and energy efficiency, and optimizing structural maintenance.

## Introduction

Compared to other steels, the H-beam offers a more sensible strength-to-weight ratio and a more optimized cross-sectional area distribution. Because of how much its cross-section resembles the English letter "H", it is known as a "H-beam". Therefore, in practical engineering, the detection of the stress state of H-beams has a significant effect on the safety and stability of construction. To monitor the safety and stability of H-beams, many scholars have conducted relevant research on monitoring their damage^1^. H-beams are widely used in the steel of reinforced concrete beams (SRC). Tong studied the fatigue failure mode of an H-beam in an SRC^2^ and found that the fatigue failure mode of this steel in an SRC was essentially the same as that of pure steel beams. Sungmo and Choi conducted research on ways to enhance the stability of H-beams^3^. In their study, experiments were conducted on different specimens, such as end reinforced steel plates, steel bars, and reinforced studs. Jeong et al.^4^ studied the ductility of composite and conventional H-beams. For example, for a composite H-beam composed of an H-beam and glulam^5^, limit state analysis was used to analyze the load–displacement hysteresis curve, and it was found that the ductility of the composite H-beam was 138% higher than that of the conventional H-beam. Some researchers have also studied the seismic performance of H-beams after corrosion^6^, shear force of steel beams^7^, and axial compressive bearing capacity^8^ and found that the higher the degree of corrosion, the worse the seismic performance. The weaker the beam’s shear force, the lower the axial compressive bearing capacity. The hysteresis behavior of H-beams has a significant effect on the stability judgment of H-beams. Zhao^9^, Qi^10^, and others have studied the hysteresis behavior of H-beams, found the relationship among them, and proposed related models. The effect of temperature on the properties of the H-beam is also important. Choi investigated how resistance and failure temperature affected the way H-beam compression members failed^11^. The results showed that the resistance was close to the yield force and not EC3. The elastic buckling load was evaluated at the failure temperature obtained from a stress-dependent test. Several factors can affect the mechanical properties of H-beams. With a rise in the opening ratio, the corresponding bearing capacity reduction coefficient became more noticeable for specimens with dense web openings, according to Kong^12^'s study of the mechanical characteristics of perforated welded H-beam short columns under axial compression. Many methods have been utilized to detect damage, such as the use of ultrasonic technology to study fatigue damage behavior, which can reveal the severity of damage through strain signals^13^. In addition, infrared thermal imaging^14^ was used to detect and locate underground steel damage^15^. With the constant advancement of science and innovation, nondestructive testing (NDT) has bit by bit been applied in different fields. A visual detection method of steel structure defects based on laser ultrasonic guided wave & piezoelectric metal wire sensor is proposed by Kang et al.^16^ To increase the strength of steel, Bahrampoor^17^ used a diagonal eccentrically braced frame. He conducted simulations using finite element analysis and found that by replacing carbon steel of equal strength on the chain links with other low-strength steels, its performance could be fundamentally improved without causing any global or local instability to its links. Knoedel^18^ studied the dynamic behavior of X-braces and found that the natural frequencies in the classical sense could not be defined because the stiffness of the system changed during cyclic motion. This also affected the maximum amplitude produced under harmonic excitation. In terms of steel structure health monitoring, the results of Liu's^19^ investigation into the use of the Internet of Things health monitoring system in this area revealed that the time series model created by the system could accurately and definitely anticipate future trends and laws of strain data. Lopato^20^ introduced an application of a microstrip antenna sensor in the distortion supervision of bending steel structure, but this method was limited to planar microstrip sensors to analyze the influence of patch sensor curvature on the resonant frequency during the bending process. Dinkler^21^ introduced a concept for online structural health monitoring, integrating piezoelectric ceramic elements as actuators and sensors into thin steel plates, allowing them to excite the structure and measure the dynamic system response. To find adhesive in CFRP steel constructions, Li^22^ employed a guided wave-based structural health monitoring method. The received signal's time of flight (TOF) for the first arrival wave packet and the severity of the debonding injury were shown to be linearly correlated. However, the research on the health monitoring of H-beams after adding diagonal braces is insufficient.

As a crucial element of load-bearing structures, H-shaped steel can be monitored for stress states to detect potential structural issues and anomalies effectively. This is of significant importance in ensuring the stability and safety of the structure. However, H-shaped steel structures are often exposed to complex environmental conditions such as high temperatures, humidity, and corrosive media that can potentially interfere with detection equipment and sensors, leading to measurement errors. Moreover, the current techniques and methodologies for measuring may face challenges when dealing with intricate shapes and surfaces that are difficult to access in H-shaped steel. Previous research has yielded some advancements; however, unexplored areas remain, particularly structural health monitoring for H-shaped steel beams reinforced with diagonal braces. The present study proposes a health monitoring approach utilizing piezoelectric ceramic sensors for H-shaped steel structures with diagonal braces, and investigates the stress conditions of H-shaped steel with different web heights and pressures, and quantitatively evaluates the H-shaped steel support structures under different web heights and pressures using energy indicators. The proposed health monitoring method for H-shaped steel with diagonal braces enables nondestructive testing, offering a novel research perspective for monitoring and evaluating H-shaped steel in practical engineering applications. The present paper is structured as follows: Sect. 3 introduces the monitoring principles and algorithms employed in the experiment, while Sect. 4 provides an overview of the entire experimental setup, procedures, and other relevant details. In Sect. 5, we discuss the experimental results, followed by numerical simulations and their validation in Sect. 6. Finally, Sect. 7 summarizes our research findings and proposes future research prospects and recommendations.

## Principle and algorithm of experimental monitoring

### Principle of studying the stress state of H-beams using a PZT sensor

**Figure 1:**
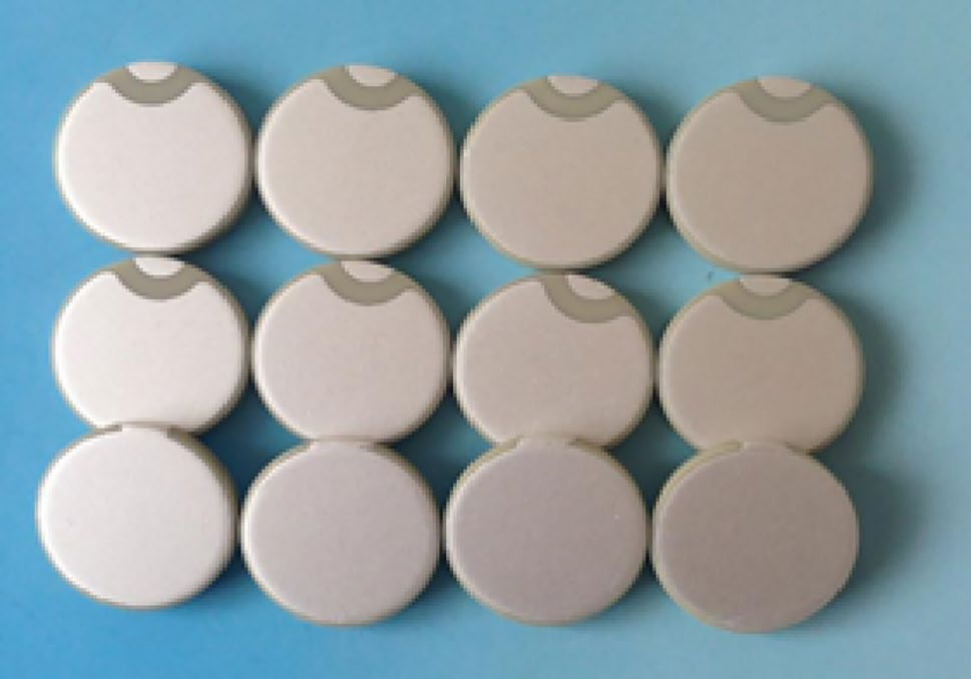
Piezoelectric ceramic sheet.

In this experiment, lead zirconate titanate was used in the piezoelectric ceramic material, as shown in Fig. 1. As observed in previous studies, piezoelectric ceramics are widely used as sensors for measuring strain and stress waves^23^. Piezoelectric ceramics can also be used as transducers for damage detection in structures^24^. Piezoelectric materials should have good conversion properties, such as high piezoelectric constant, dielectric constant^25^, resistivity, mechanical strength, and rigidity. It has good temperature and humidity stability, and a high Curie point is required to obtain a wide operating temperature range. Moreover, the piezoelectric properties do not change with time. The parameters of PZT used in this study are shown in Table 1.

**Table 1:** Relevant material properties of PZT-5H.

Material property	Value
Density (kg/m^3^)	7600
Electromechanical coupling coefficient (kP)	0.6
Piezoelectric constant d33 (C · N–1)	4 × 10–10
Poisson's ratio	0.35
Mechanical quality factor (Qm)	80
Relative permittivity (εr33/ε0)	1600
Dielectric loss (tanδ)	0.025
Curie temperature (°C)	360
Elastic modulus (109 N/m^2^)	117

In this experiment, a PZT sensor was used to analyze the force of the diagonally braced H-beam. A diagonally braced H-beam of the same size was selected, loads of different intensities were applied from the top, and a piezoelectric ceramic sheet was bonded to the diagonally braced H-shaped steel web and connected with the signal receiver through a wire. When loads of different intensities were applied on the diagonally braced H-beam, the signal receiver received different signals; the PZT piece was connected to the upper, middle, and lower positions of the diagonally braced H-beam.

The principle of stress state monitoring via piezoelectric signals is based on the utilization of the piezoelectric effect to measure an object's stress and strain state. This is due to the fact that piezoelectric materials exhibit said effect, generating electric charges or signals when subjected to force or stress. This principle allows for the use of piezoelectric ceramic materials as sensors, indirectly reflecting a material's stress state by monitoring changes in its piezoelectric signals. In the proposed health monitoring algorithm, to test the sensitivity of the propagation of the test wave signal between the diagonally braced H-beams and the degree of load, the time-domain analysis method used in this study is based on the change of time and signal values. With an increase in the web height and pressure of the diagonally braced H-beam, the signal value received by the signal receiver also changes; this change has a certain regularity.

### Essential principles of time domain analysis

In the diagonally braced H-beam used in this experiment, the transmission of the test wave signal is very susceptive to the force of the H-beam and can produce obvious changes. In this study, time domain analysis is used to research the signals accepted by piezoelectric ceramic sensors. By calculating the energy of the signal, the stress and strain of the diagonally braced H-beam can be measured. In the proposed algorithm, *E* is defined as the signal energy index, $X_{i}$ represents a set of discrete data of the signal obtained by the sensor during a given sampling duration at time i, and $x_{ij}$ represents the sensor voltage value at the jth sampling point at time i. During each sampling period, there were altogether m sampling points, which facilitated the calculation. $S_{i}$ is equal to the integral area surrounded by the signal i curvilineal registered on the time i and the x axis. $X_{i}$ is represented by Eq. (1):

1 $$X_{i} = (x_{i1} ,x_{i2} ,\ldots,x_{im} ),\quad (i = 1,2,\ldots)$$

To monitor the stress state of the diagonally braced H-beam, the energy indicator is defined as

2 $$E_{i} = S_{i}^{2} = \left[ {\int {\left| {x_{ij} } \right|dt} } \right]^{2} ,\quad (j = 1 \, \ldots \, m)$$

where $E_{i}$ is an index to monitor the force change of the H-beam of the diagonal brace, and the wave transmission energy detected by the PZT sensor can be displayed by this index. And the stress and strain of the bracing H-beam can also be detected. In this experiment, when the diagonally braced H-beam was stressed, the stress and strain at the web of the diagonally braced H-beam were large, and the energy signal detected by the PZT sensor was also large. The stress condition of the diagonally braced H-beam can be studied based on its sensor response. However, in experiments, the performance of the PZT sensor was significantly affected by the ambient temperature. This is mainly due to the change in the piezoelectric coefficient and dielectric constant of PZT and the performance of the PZT packaging materials with temperature. With an increase in temperature, the mode frequency and compressive stress in the thickness direction of the PZT also increase. However, this test mainly studied the stress of the diagonally braced H-beam. Accordingly, to decrease the impact of temperature change on the sensor signal, the surrounding temperature was kept up with at 20 °C during the trial.

## Experimental overview

### Experimental setups

**Figure 2:**
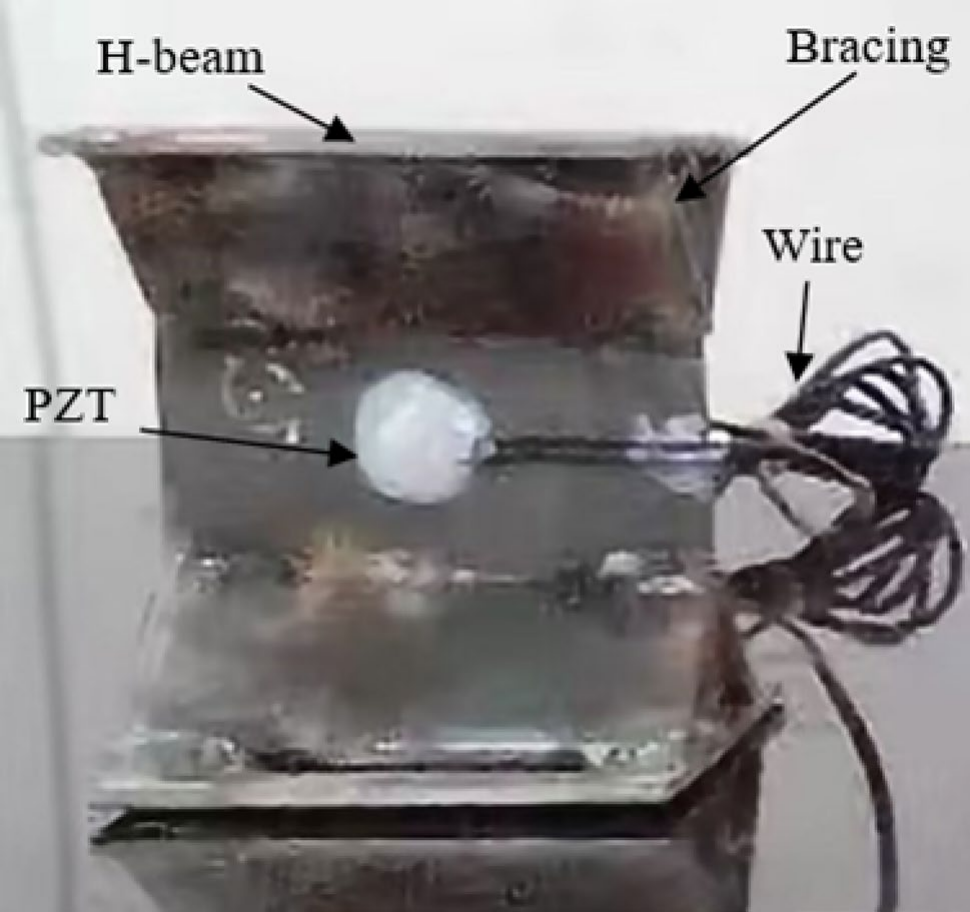
Schematic of the experimental specimen.

**Figure 3:**
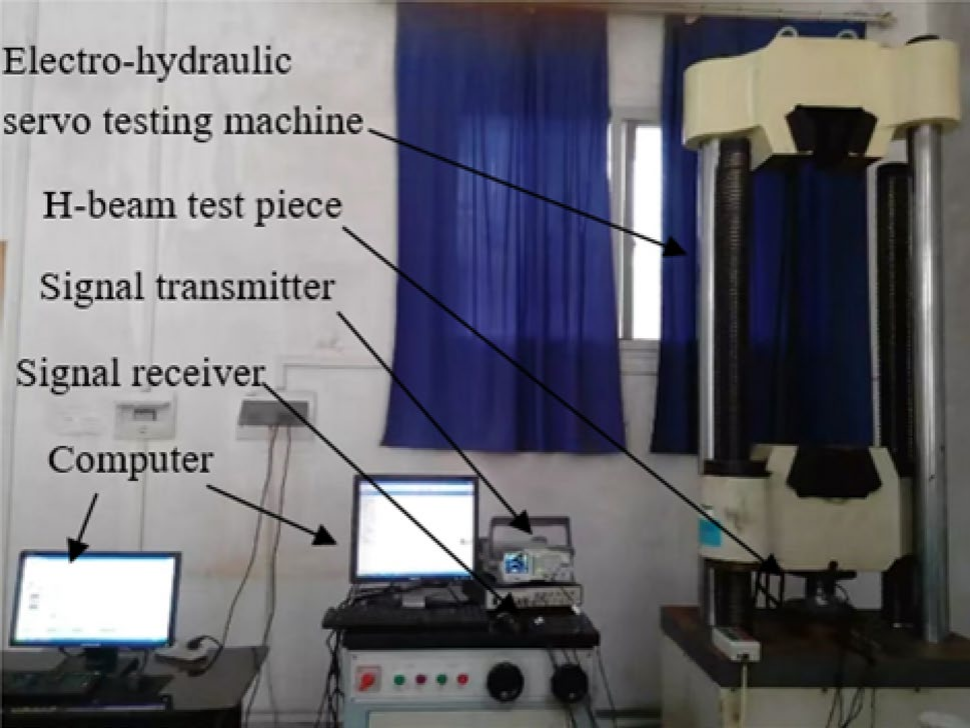
Experimental setup.

As shown in Fig. 2, the width, length, web width, and height of the diagonally braced H-beam flange plate were 10 cm, while its thickness was 0.35 cm. Professional welders were required to weld the structure, and the diagonal brace was 0.3 cm in thickness. Welding started from a distance of 1 cm from the edge of the upper and lower flange plates, connected the webs with each other and the upper and lower diagonal braces to the webs at a distance of 3 cm from the upper and lower flange plates. As shown in Fig. 2, the PZT sheet was bonded to the center of the web using AB glue. Figure 3 is the experimental device diagram; the signal is recorded by INV306U signal acquisition instrument. The PZT piece was connected to the signal receiver through a wire, the sampling frequency of the signal acquisition device is 7160 Hz and the sampling time is 0.08 s, and the output channel precision is 16 bits, and the resolution is 10 ns. The PZT as a sensor receives real-time signals and is connected to the diagonally supported H-beam. The positions of the PZT pieces are shown in Fig. 2. During the experimental process, the H-shaped steel specimens were positioned onto an electro-hydraulic servo testing machine and subjected to static loading based on predetermined experimental parameters. The electro-hydraulic servo testing machine has a test force measurement range of 2–100% of maximum test force, with constant stress control ranging from 2–60 N/mm^2^ s^−1^, constant displacement control ranging from 0.5–50 mm, and constant strain control ranging from 0.00025/s–0.0025/s. When the size of the H-beam of the diagonal brace was fixed, loads of different intensities were simultaneously applied on the top of the H-beam of the diagonal brace simultaneously. By changing the intensity of the load, the shear lag can be studied by observing the changes in the web shear flow. When subjected to the stress generated by the pressure, the diagonal brace H-beam generates strain, The signal transmitter emits a sinusoidal signal through the PZT patch connected to the left side of the H-shaped steel, while the signal receiver detects the same signal via the PZT patch connected to the right side of the H-shaped steel, and the signal receiver receives a different signal. The sine wave emitted by the signal transmitter has a frequency of 1000 Hz and an amplitude of 100,000 mV. The stress condition of the diagonally braced H-beam can be observed by studying the trend of the signal change.

### Experimental steps

**Figure 4:**
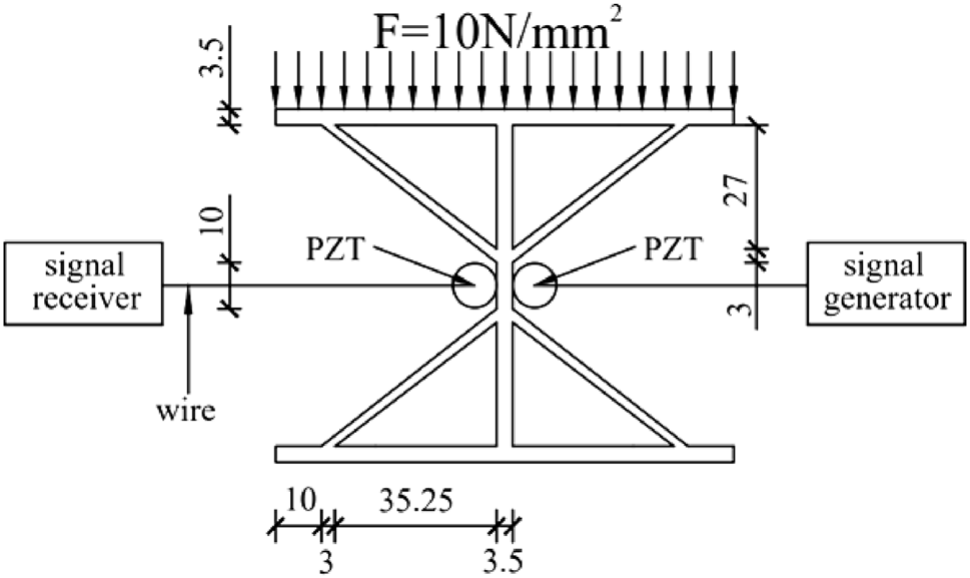
Schematic of the experimental setup for Group A (unit: mm).

**Figure 5:**
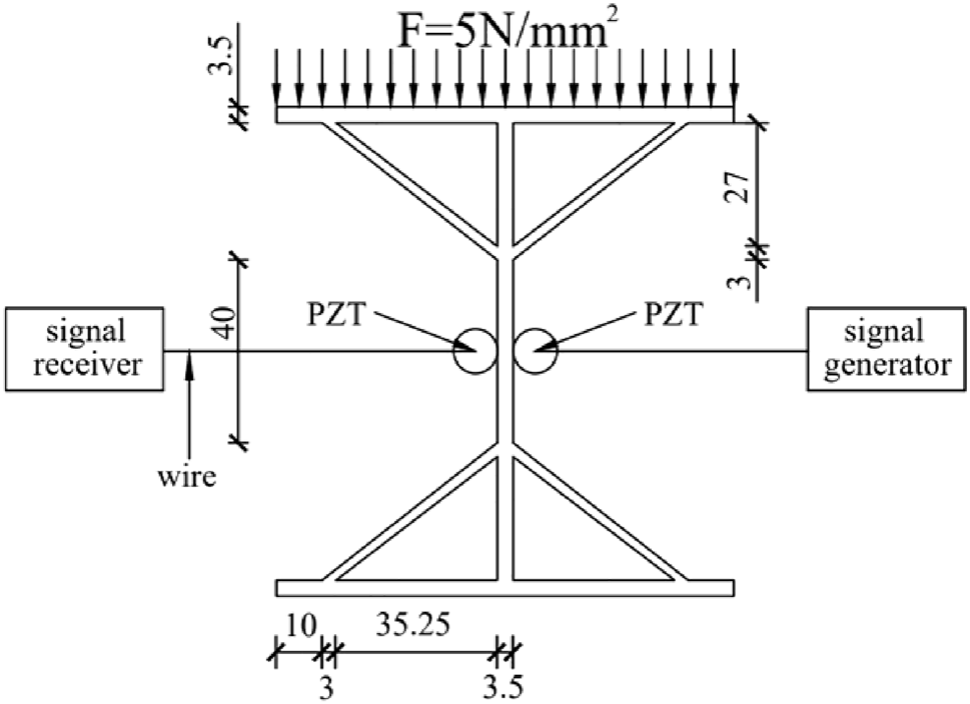
Schematic of the experimental setup for Group B (unit: mm).

In this study, it was found that, owing to the difference in the web height and applied pressure of the diagonally braced H-beam, the stress and strain differed. Accordingly, in this experiment, two groups of diagonally braced H-beams with different variables were selected: Groups A and B. It was assumed that the distance between the other end of the diagonal brace and the edge of the flange plate was 1 cm. In this experiment, the H-beams with different web heights displayed different strains under the same pressure, while those with same web heights displayed different strains under different loads, as shown in Table 2. The uniform thickness of the diagonal braces of Groups A and B was 0.3 cm. In Group A, the fixed pressure was 10 N/mm^2^, and the web heights were 7, 10, and 13 cm. In Group B, the web height was fixed at 10 cm, and the pressures were 5, 10, and 15 N/mm^2^. The web heights and load conditions are listed in Table 2. The design objectives of Groups A and B are to study the mechanical behaviours of H-shaped steel under different web heights and load strengths. The design parameters for Groups A and B have been carefully selected to facilitate a direct comparison of the test specimens under identical testing conditions, thereby ensuring the reliability and accuracy of the research findings. The design parameters of Groups A and B can be effectively implemented in practical operations, considering the experimental equipment and testing conditions, thereby ensuring reliable experimental results. To ensure that the two groups of experiments are conducted an equitable situation, other experimental conditions were guaranteed to be the same, including but not limited to the part where the diagonal bracing connects the flange plate. The distance between the diagonal bracing and thickness of the flange plate were constant. Figures 4 and 5 are schematics of the experimental setup in Group A and Group B. During the experimental procedure, Group A and Group B specimens were tested under the designed load conditions while simultaneously monitoring the signal variations received by the piezoelectric ceramic sensors. The experiment maintained consistency in the conditions, design, and research content. Furthermore, the experimental conditions closely replicated the loading conditions of diagonally braced H-shaped steel in engineering applications. Through the experimental design, the influence of different web heights and load strengths on the stress state of H-shaped steel can be investigated, thus verifying the rationality of the research objectives.

**Table 2:** Testing conditions for Groups A and B.

	Case	1	2	3
Group A	Web height	7 cm	10 cm	13 cm
Group B	Load	5 N/mm^2^	10 N/mm^2^	15 N/mm^2^

## Experimental results and discussion

**Figure 6:**
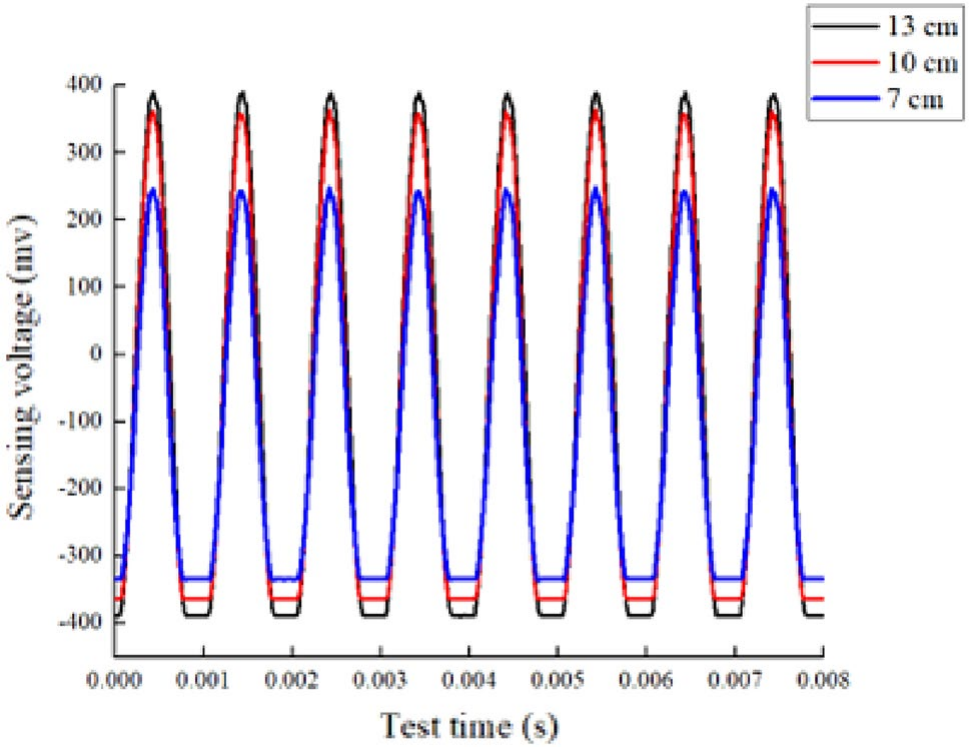
Group A signal response plot.

**Figure 7:**
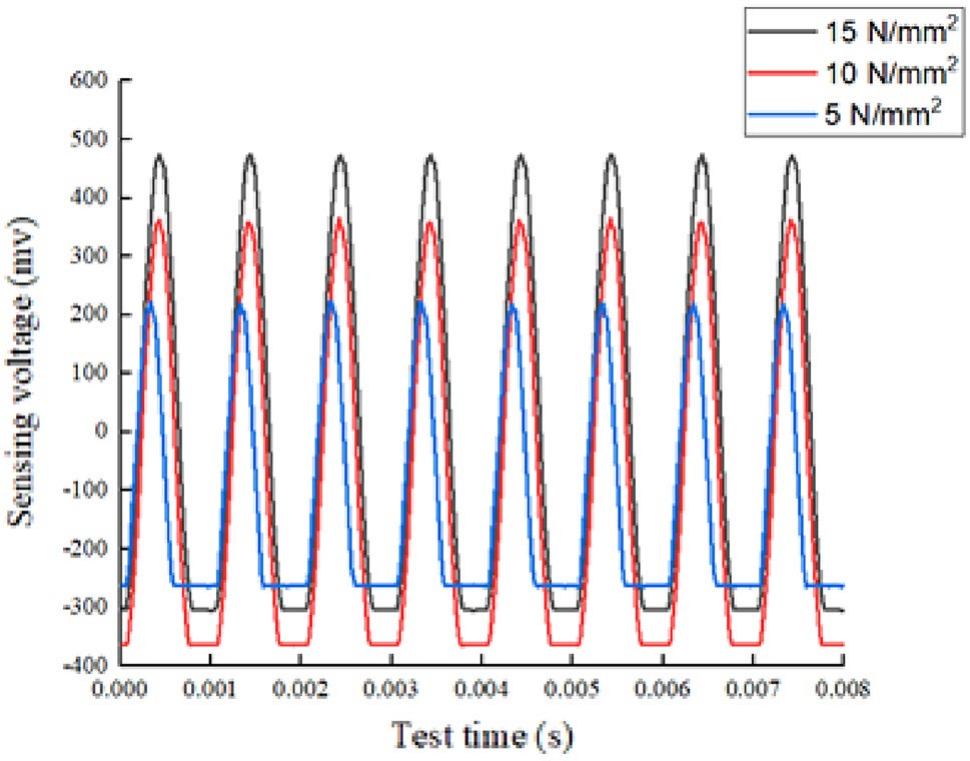
Group B signal response plot.

Figures 6 and 7 show the results of time domain signal response of Group A and Group B respectively. The curves in the two figures are the signal response of the transducer when the bracing H-beam is compressed. According to the formulas (1) and (2), the energy index was calculated for all experimental cases in the experimental grouping, and the results showed that the index value increased with the increase of load and web height.

In the case of Group A, when the web height was 7 cm, the measured time-domain waveform signal amplitude was 247.5176 mv and the time-domain waveform energy index was 25.8617 v^2^; when the web height was 10 cm, the measured time-domain waveform signal amplitude was 364.4203 mv and the time-domain waveform energy index was 34.7189 v^2^; and when the height of the web was 13 cm, the time-domain waveform signal amplitude was 389.526 mv and the time-domain waveform energy index was 38.8497 v^2^. It can be seen from the data that with every 1 cm increase in the height of the web, the amplitude of the time-domain waveform signal increased by approximately 9.6% and the energy index of the time-domain waveform increased by approximately 8.4%.

In the case of Group B, when the pressure value was 5 N/mm^2^, the measured time-domain waveform signal amplitude was 222.13 mv and the time-domain waveform energy index was 19.2972 v^2^; when the pressure value was 10 N/mm^2^, the measured amplitude of its time-domain waveform signal was 364.4203 mv and the time-domain waveform energy index was 34.7189 v^2^; and when the pressure value was 15 N/mm^2^, the time-domain waveform signal amplitude was 473.734 mv, and the time-domain waveform energy index was 35.7045 v^2^. It can be seen from these data that when the pressure value was less than 10 N/mm^2^, the energy index increased by approximately 15.98% for every 1 N/mm^2^ increase in pressure on average. When the pressure value was greater than 10 N/mm^2^ and less than 15 N/ mm^2^, the energy index increased by roughly 1% for every 1 N/mm^2^ increase in pressure on average.

**Figure 8:**
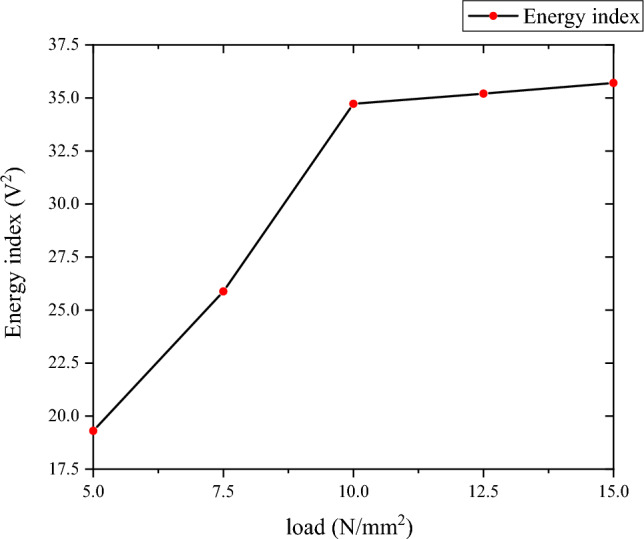
Energy index of different load.

**Figure 9:**
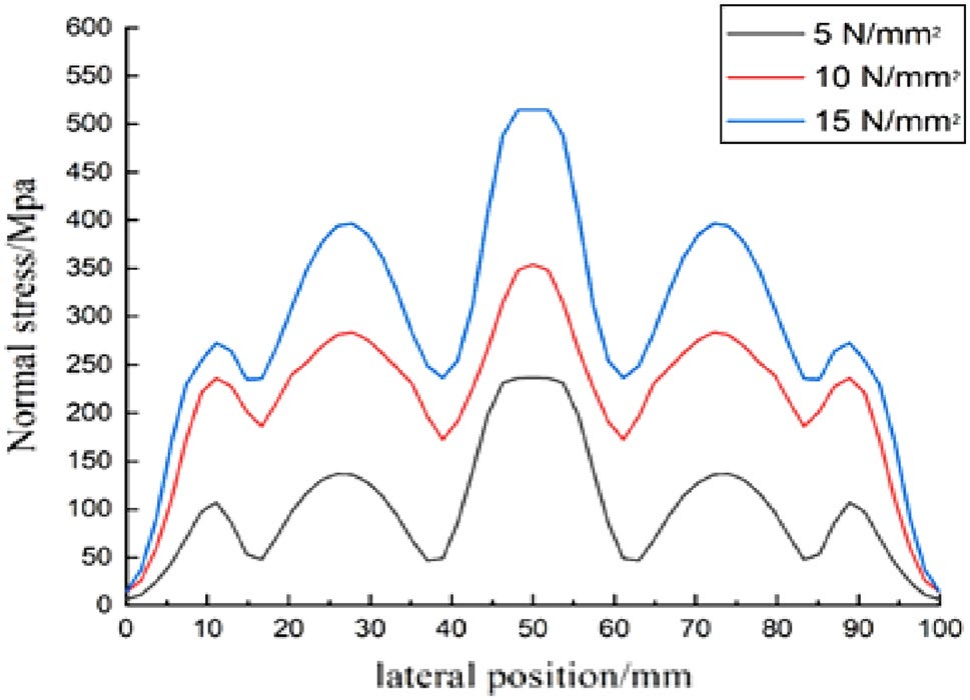
Transverse distribution of normal stress on the roof.

According to the analysis of the experimental results, the amplitude and energy index of the energy signal increase with the increase in the web height of the diagonally braced H-shaped steel and the applied load intensity. The amplitude and energy index increase are attributed to the constraints imposed on the diagonally braced H-shaped steel h-beam under varying web plate pressure and height conditions. With the increase in web height and applied load intensity, the stress at the PZT patch's bonding location intensifies, reducing atomic gaps and resulting in a more secure bond. As a result, the signal emitted by the transmitter experiences less attenuation during propagation, leading to a significant reduction in PZT attenuation and an increase in the amplitude of the received signal. The correlation between piezoelectric signals and stress is illustrated in Fig. 8. As depicted in the figure, increased stress leads to a corresponding augmentation of piezoelectric signals. The correlation between top load and shear hysteresis behaviour is illustrated in Fig. 9. As demonstrated, an increase in top load leads to a more pronounced shear hysteresis phenomenon.

By means of real-time monitoring of the provided signal data, nondestructive testing, and monitoring can be maximized for the structure, which is highly significant in researching structural health monitoring. The proposed method enables real-time damage detection and accurate assessment of deformation severity in diagonally braced H-shaped steel beams, thus offering a novel research perspective for nondestructive structural health monitoring. During the study, experimental results may be influenced by environmental conditions such as humidity and temperature, which can introduce errors. Additionally, the choice of testing equipment can also impact the experimental outcomes.

## Experimental simulation and numerical verification

**Figure 10:**
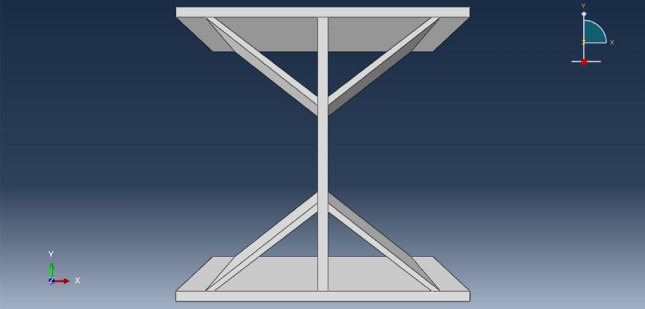
Sketch of the finite element calculation model.

The finite element analysis, a widely employed numerical method for analysis, can be utilized to model structures and predict the structure's stress, strain, and response. To further study the influence of load strength and web height on the stability of diagonally braced H-beams, and validate t the reliability and effectiveness of our experiment, we conducted finite element analysis and numerical simulations using Abaqus. The relevant patterns were explored through the results obtained from these simulations. Figure 10 shows the schematic of the calculation model. To enhance the validation of experimental results and ensure the complementarity between simulation and experimentation and consistency in outcomes, the finite element model parameters were set to be identical to those utilized in experiments. The geometric dimensions and design parameters of the finite element model were also matched with those of the laboratory model, the finite element model comprised an H-beam and diagonal braces. The specific geometric dimensions are as follows, and the diagonally braced H-beam flange plate had a width, length, and web height of 10 cm, thickness of 0.35 cm, bracing thickness of 0.3 cm, and width of 6 cm. The connecting position of the bracing and flange plate is 1 cm away from the edge of the flange plate. The connection position between the upper and lower diagonal braces and the web was 3 cm away from the flange plate, and the non-damage data was set to the healthy state. The model parameters are as follows: the elastic modulus E is 200 GPa, the Poisson's ratio is 0.33, the density is 7.85 g/cm^3^, the material linear thermal expansion coefficient is 1.20E−5/°C, the tensile strength is 450 MPa, and the yield strength is 235 MPa. By utilizing finite element analysis, the stress–strain responses of diagonally braced H-shaped steel can be determined under various design loads, while simultaneously validating experimental results.

**Figure 11:**
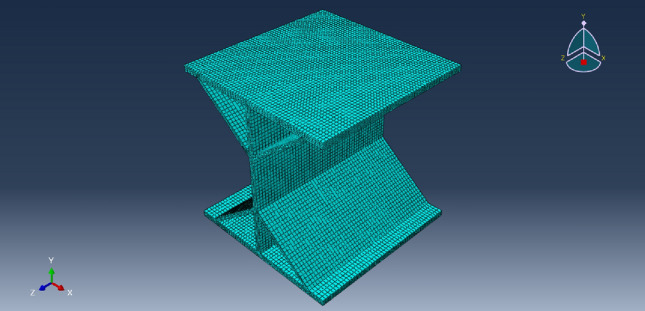
Model meshing.

**Figure 12:**
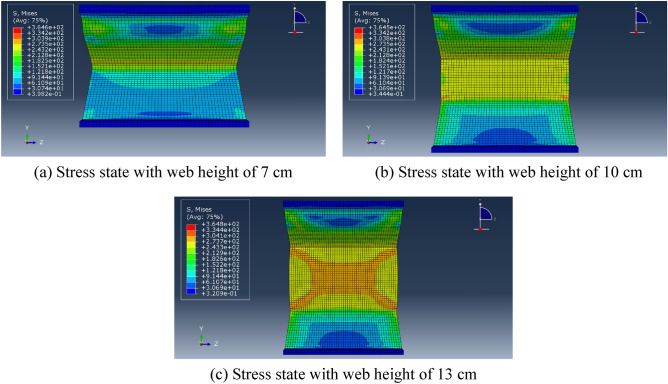
Group A finite element stress analysis results.

**Figure 13:**
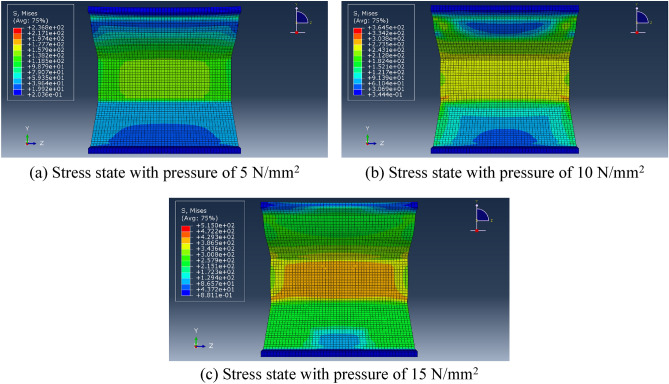
Group B finite element stress analysis results.

**Figure 14:**
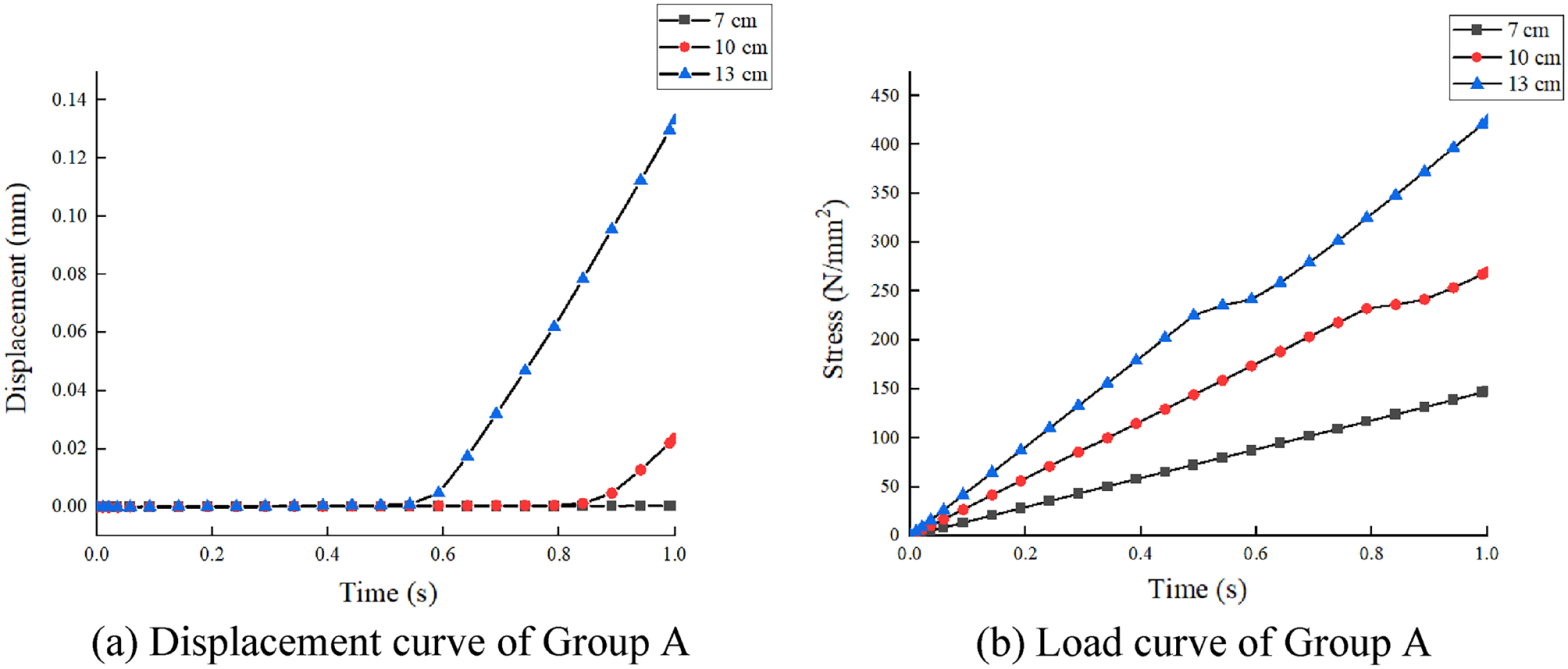
Group A load and displacement curves.

**Figure 15:**
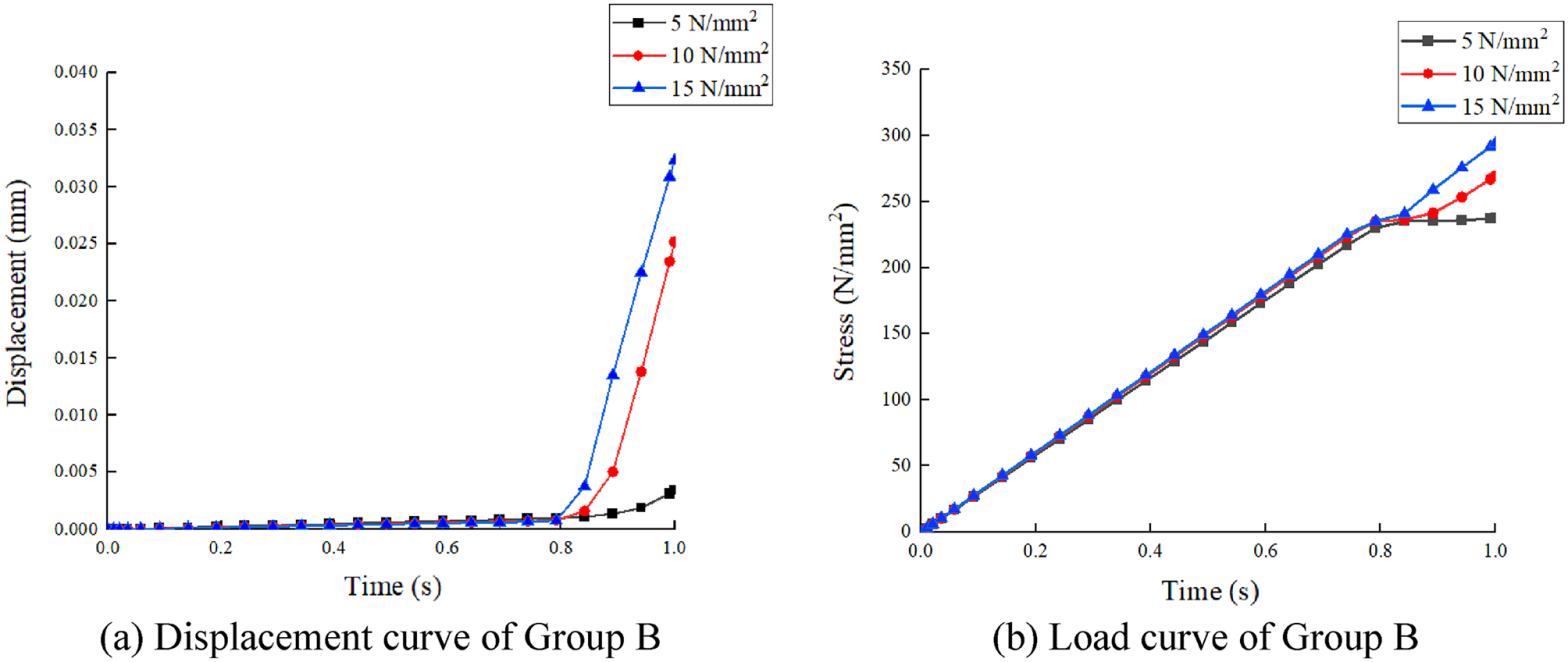
Group B load and displacement curves.

In the finite element modeling process, to meet the conditions of the real experiment, the loading of loads in finite element analysis commences at zero and is incrementally raised to the predetermined value, pressure was applied to the upper flange plate of the diagonally braced H-beam up to the design load, and constraints in the x-, y-, and z-directions were added to the lower flange plate. In Groups A and B, each group consisted of three models. The models in Group A were subjected to a load of 10 N/mm^2^, with varying web heights of the beam at 7 cm, 10 cm, and 13 cm, respectively. In contrast, Group B models featured an inclined brace h-beam with a fixed web height of 10 cm and applied loads ranging from 5 N/mm^2^ to 15 N/mm^2^. To truly simulate the force state and ensure the transmission of force, the model was divided into four layers when meshing, as shown in Fig. 11. Figures 12 and 13 show the stress–strain state of the diagonally braced H-beam under different strength loads and web heights. The calculation results of the ABAQUS finite element analysis are shown in Figs. 14 and 15. The stress and displacement values presented in Figs. 14 and 15 were obtained from the front centre position of the diagonally braced H-shaped steel, providing a precise representation of its structural behaviour. A piezoelectric sheet was pasted on the web of the diagonally braced H-beam. It can be seen from the figure that the stress and strain generated by the diagonally braced H-beam increased with the continuous increase in the height of the web and pressure. At the same time, it can be observed from the finite element model that the stress in the diagonally braced H-shaped steel increases with the increase in pressure and the height of the web plate. The simulation results indicate that in Group A, the stress and displacement of diagonally braced H-shaped steel exhibit an increasing trend with the height of the web plate. This is due to the fact that as the height of the web plate increases, so does its area, resulting in a rise in stress. And the displacement also increases with the increase in the height of the web plate of the diagonally braced H-shaped steel. The stress and displacement of the inclined brace H-shaped steel in group B also exhibit an increasing trend with the rise in load strength. This can be attributed to the fact that during the elastoplastic deformation stage, both stress and displacement of the material escalate as load strength increases, by comparing the experimental and numerical simulation data, it can be observed that the stress experienced by the diagonally braced H-shaped steel increases with the increase in height and the applied load strength.

## Conclusion

To monitor the change in the force of the H-beam under the condition of increasing diagonal braces and achieve nondestructive structural health monitoring to the greatest extent possible, an electro-hydraulic servo testing machine was utilized in this study to simulate repetitive loading responses. A method was proposed for achieving real-time monitoring by analyzing the energy signal response of piezoelectric ceramic sensors. Two cases were studied: the same height of the web of the diagonally braced H-beam and different pressures, and the same pressure of the diagonally braced H-beam but different web heights. It can be seen from the experimental study that under the working conditions of Group A, with an increase in the height of the H-beam web of the diagonal brace, the signal amplitude and energy index received by the PZT sensor increased accordingly. An average increase of 1 cm in the height of the web increased the amplitude of the time-domain waveform signal by roughly 9.6% and the energy index of the time-domain waveform by roughly 8.4%. Similarly, under the working conditions of Group B, the signal amplitude and energy index increased with an increase in the pressure on the H-beam of the diagonal brace. When the pressure was less than 10 N/mm^2^, the energy index increased by roughly 15.98% for every 1 N/mm^2^ increase in pressure on average. An average increase of 1 N/mm^2^ in pressure will increase its energy index by roughly 1%. With the increase in pressure and web height, the strain generated by the H-beam of the diagonal brace and the mechanical deformation of the PZT sensor increased; thus, the signal amplitude and energy index received by the sensor also increased. The statement above implies that the stress–strain amplitude of diagonally braced H-shaped steel exhibits a positive correlation with the increase in web height and applied load intensity.

The energy index value also provides a quantitative assessment of the degree of damage of the diagonally braced H-beam. The degree of damage of the diagonally braced H-beam can therefore be determined. According to the research results, ensuring the safety and reliability of important load-bearing structures holds significant value through strict control over their dimensions and force magnitude in practical applications. The health monitoring method proposed in this study facilitates real-time monitoring of the health status of H-shaped steel structures in practical engineering applications, thereby ensuring the safety and stability of these structures. Despite the significant findings and results obtained in this study, several avenues still require further exploration and investigation. In future research, a more comprehensive examination of health monitoring for H-shaped steel structures in complex environments can be conducted to elucidate more efficient monitoring methodologies.

## Data Availability

The experimental data used to support the findings of this study are included within the article.
